# Are personality disturbances in anorexia nervosa related to emotion processing or eating disorder symptomatology?

**DOI:** 10.1186/s40337-015-0071-1

**Published:** 2015-10-01

**Authors:** Andrea Phillipou, Caroline Gurvich, David Jonathan Castle, Susan Lee Rossell

**Affiliations:** Department of Optometry & Vision Sciences, The University of Melbourne, Melbourne, Australia; Department of Psychiatry, The University of Melbourne, Melbourne, Australia; Department of Mental Health, The Austin Hospital, Melbourne, Australia; Monash Alfred Psychiatry Research Centre, Melbourne, Australia; Department of Psychiatry, St Vincent’s Hospital, Melbourne, Australia; Faculty of Health Sciences, Australian Catholic University, Melbourne, Australia; Brain and Psychological Sciences Research Centre, Swinburne University of Technology, Melbourne, Australia; Department of Mental Health, St Vincent’s Hospital, 46 Nicholson St, Fitzroy, VIC 3065 Australia

## Abstract

**Background:**

Anorexia Nervosa (AN) is a psychiatric illness associated with a number of personality disturbances. However, whether these personality characteristics are related to eating disorder symptomatology or emotion regulation is unclear. The aim of this study was to investigate these relationships.

**Results:**

Twenty-four individuals with AN and 25 age- and premorbid intelligence-matched controls completed the Personality Diagnostic Questionnaire, and scores were correlated with measures of emotionality and negative mood states, and eating disorder symptomatology. AN was associated with increased scores on schizoid, borderline, avoidant, dependent, obsessive compulsive, negativistic and depressive personality dimensions, relative to controls. In AN, eating disorder symptomatology did not significantly correlate with scores on any personality dimension. However, a number of personality characteristics were found to correlate with negative mood states.

**Conclusions:**

The findings suggest that personality disturbances in AN are not related to disorder-specific symptoms, but are related to negative mood states.

## Background

Anorexia Nervosa (AN) is a psychiatric illness characterised by an intense fear of weight gain, low body weight, and a disturbance in the experience of one’s own body shape or weight. The condition is also frequently associated with a number of personality traits, including perfectionism [1–5], rigid thinking patterns [6] and obsessive compulsive behaviours and thoughts [7].

Relatedly, AN has been associated with high levels of comorbid obsessive compulsive personality disorder, as well as avoidant, borderline and dependent personality disorders [8–11]. Personality disorders are related to poor emotion processing and negative mood states [12, 13], which are similarly reported in AN [14, 15]. Thus, it is unclear whether personality disturbances in AN interact with eating disorder symptomatology, or whether they relate to difficulties in emotion regulation. Thus, the aim of this study was to investigate these relationships in AN. Furthermore, given the lack of current treatment options for AN [16], this research is warranted to determine which personality variables are associated with mood regulation or eating disorder symptomatology to help guide the development of more tailored and effective interventions for AN.

It was hypothesised that the AN group would demonstrate higher scores on a number of personality disturbances relative to healthy controls, namely, obsessive compulsive, avoidant, borderline and dependent personality disorders. Given the lack of research investigating relationships of other psychological processes with personality disturbances in AN, the primary aim of this study was exploratory, to examine whether personality disturbances in AN relate to mood states or emotion regulation within the self (i.e. alexithymia) and eating disorder symptomatology.

## Method

This study was approved by the human research ethics departments at The University of Melbourne, Swinburne University of Technology, The Melbourne Clinic, The Austin Hospital and St Vincent’s Hospital; all in Melbourne, Australia. Informed written consent was obtained from all participants. The authors assert that all procedures contributing to this work comply with the ethical standards of the relevant national and institutional committees on human experimentation and with the Helsinki Declaration of 1975, as revised in 2008.

### Participants

Participants were 24 right-handed females with AN and 25 healthy controls (HC) matched for age and premorbid intelligence quotient (IQ) to ensure comparability between groups. HCs were recruited through public advertisements, whereas AN participants were recruited through public advertisements; the Body Image and Eating Disorders Treatment and Recovery Service at the Austin and St Vincent’s Hospitals; and The Melbourne Clinic; all in Melbourne, Australia. The Austin and St Vincent’s Hospitals are public hospitals accepting referrals from Northern, Eastern and Hume regions of Melbourne. The Melbourne Clinic is a private mental health service, and unlike the public health services, only accepts patients on a voluntary basis.

All participants were English speaking and had no history of significant brain injury or neurological condition. Controls were required to have no history of an eating disorder or other mental illness. The Mini International Neuropsychiatric Interview, 5.0.0 (MINI) [17] was used to screen all participants for major Axis I psychiatric disorders according to the Diagnostic and Statistical Manual of Mental Disorders (DSM-IV). It was also used to confirm diagnoses of AN, with the exception of the amenorrhea criterion which is no longer included in the current DSM-5. AN was required to be the primary diagnosis of the AN group. AN participants with comorbid psychiatric conditions, other than psychotic conditions, were not excluded as this would not have represented a typical AN sample. The following comorbid conditions were identified in the AN group: current depressive episode (n = 17), dysthymia (n = 14), current manic/hypomanic episode (n = 2), current panic disorder (n = 3), social anxiety disorder (n = 8), obsessive compulsive disorder (n = 12), posttraumatic stress disorder (n = 4), alcohol dependence (n = 2), substance dependence (n = 1), bulimia nervosa (n = 3), generalised anxiety disorder (n = 17).

Premorbid intelligence was estimated using the Wechsler Test of Adult Reading (WTAR) [18] and was used to ensure all participants had an IQ over 80, to ensure they could adequately comprehend the study instructions and correctly complete the questionnaires. Eating disorder symptomatology was investigated with the Eating Disorders Examination Questionnaire (EDE-Q) [19] (Table 1), and negative emotional states with the Depression Anxiety Stress Scale (DASS) [20]. The alexithymia construct was measured with the Toronto Alexithymia Scale (TAS-20) [21], and personality psychopathology with the Personality Diagnostic Questionnaire (PDQ-4) [22]. The PDQ-4 allows for the examination of continuous symptom scores for each personality disorder and categorical scoring (i.e. presence or absence) of clinically significant personality disorders.

**Table 1:** Participant information

	AN		HC		
	M	SD	M	SD	*p*
Age	23.07	6.88	22.67	3.19	0.798
Premorbid IQ	104.67	8.19	105.60	7.00	0.670
BMI	16.52	1.14	22.40	3.59	0.001
Illness duration	6.67	7.66	–	–	–
Age of illness onset	16.04	3.50	–	–	–
EDE-Q restraint	3.93	1.42	0.43	0.40	0.001
EDE-Q eating concern	3.78	1.24	0.20	0.20	0.001
EDE-Q shape concern	5.01	0.90	0.99	0.59	0.001
EDE-Q weight concern	4.50	1.41	0.42	0.47	0.001
EDE-Q global score	4.30	1.12	0.60	0.43	0.001
DASS depression	25.08	12.41	1.08	1.29	0.001
DASS anxiety	16.00	9.48	1.88	2.13	0.001
DASS stress	24.92	10.23	3.78	2.78	0.001
TAS-20 difficulty identifying feelings	22.71	5.76	10.65	3.70	0.001
TAS-20 difficulty describing feelings	18.43	2.74	10.64	4.05	0.001
TAS-20 externally oriented thinking	20.42	3.32	16.78	4.65	0.003
TAS-20 score	61.17	8.86	38.88	11.79	0.001

*AN* Anorexia Nervosa, *HC* healthy controls, *Premorbid IQ* standardised Wechsler Test of Adult Reading Score, *BMI* body mass index, *EDE-Q* Eating Disorders Examination Questionnaire, *DASS* Depression, Anxiety, Stress Scale, *TAS-20* Toronto Alexithymia Scale, *Age* age of illness onset and duration illness are reported in years

### Statistical analyses

Following normality checking and the removal of outliers (identified as cases lying outside more than 1.5x the interquartile range, through Tukey’s boxplots), group differences in EDE-Q, DASS and TAS-20 scores were examined with analyses of variance (ANOVAs). Scores on the PDQ-4 were not normally distributed, thus Mann-Whitney tests were utilised. The number of clinically significant personality disorders as identified by the PDQ-4 was also compared between groups with χ^2^ tests. Pearson’s correlations were also conducted, following outlier removal (as above), between PDQ scores and the other measures for AN and HC groups, these included DASS, TAS and EDE-Q. Due to the number of correlational analyses, alpha was set at 0.001 to account for multiple comparisons, and trends were identified as alpha <0.01.

## Results

AN participants scored significantly higher on total PDQ score, as well as items related to schizoid, borderline, avoidant, dependent, obsessive compulsive, negativistic and depressive personality disorders (Table 2). Whether the items reached clinical significance was also explored and the results are detailed in Table 3. AN participants were more likely than control participants to have a clinically significant personality disorder (χ^2^(1) = 22.40, p <0.001).

**Table 2:** Personality diagnostic questionnaire scores

	AN		HC				
	Median	Range	Median	Range	U	z	*p*
Paranoid	3.0	6.0	1.0	5.0	216.5	−1.7	0.087
Schizoid	2.5	6.0	0.0	4.0	102.2	−4.0	0.001
Schizotypal	2.0	4.0	1.0	5.0	234.0	−1.4	0.176
Histrionic	2.0	5.0	2.0	4.0	258.5	−0.9	0.389
Narcissistic	1.0	5.0	2.0	5.0	223.0	−1.6	0.112
Borderline	4.5	7.0	1.0	3.0	41.5	−5.2	0.001
Antisocial	1.0	5.0	1.0	2.0	222.0	−1.6	0.102
Avoidant	6.0	5.0	2.0	4.0	38.5	−5.3	0.001
Dependent	4.0	7.0	1.0	3.0	48.0	−5.1	0.001
Obsessive compulsive	5.0	7.0	3.0	5.0	158.5	−2.9	0.004
Negativistic	2.0	6.0	1.0	4.0	148.5	−3.1	0.002
Depressive	5.5	4.0	2.0	7.0	50.0	−5.1	0.001
Total PDQ score	38.0	47.0	19.0	26.0	51.5	−5.0	0.001

*PDQ* personality diagnostic questionnaire, *AN* anorexia nervosa, *HC* healthy control

**Table 3:** Number of clinically significant personality disorders

	AN	HC
Paranoid	3	0
Schizoid	4	0
Schizotypal	0	0
Histrionic	0	0
Narcissistic	0	1
Borderline	6	0
Antisocial	0	0
Avoidant	13	1
Dependent	5	0
Obsessive compulsive	13	3
Negativistic	2	0
Depressive	13	0
Any personality disorder	21	5

*AN* anorexia nervosa, *HC* healthy control

### Correlations with DASS subscales

In the AN group, significant positive correlations were found between schizoid personality scores and depression (r = 0.62, p < 0.001), avoidant personality scores and depression (r = 0.64, p < 0.001), and borderline personality and stress (r = 0.62, p < 0.001). Trends were identified between paranoid personality scores and stress (r = 0.52, p = 0.009), dependent personality scores and both depression (r = 0.56, p = 0.004) and stress (r = 0.56, p = 0.005), and between depressive personality scores and stress (r = 0.59, p = 0.002). In the HC group, no significant correlations were found between personality scores and DASS subscales. A trend was however identified between paranoid personality scores and anxiety scores (r = 0.53, p = 0.008).

### Correlations with TAS-20 subscales

In the AN group, no significant correlations were observed, however, a positive trend was found between schizotypal personality scores and the difficulty describing feelings subscale of the TAS-20 (r = 0.62, p = 0.002). No significant correlations or trends were observed between TAS-20 and personality scores for HCs.

### Correlations with EDE-Q subscales

In the AN group, no significant correlations were observed between EDE-Q subscales and the different personality constructs. However, schizoid personality scores were associated with a number of trends, including, the restraint and eating concern subscales of the EDE-Q (r = 0.59, p = 0.002; r = 0.53, p = 0.008), and global EDE-Q scores (r = 0.57, p = 0.003). In the HC group, one significant correlation was found between EDE-Q and personality scores: shape concern was positively correlated with negativistic personality scores (r = 0.64, p <0.001). A trend for higher eating concerns was also identified with greater avoidant personality scores in HCs (0.58, p = 0.003).

## Discussion

The aim of this study was to examine personality disturbances in AN, and their relationship to eating disorder symptomatology, negative mood states and alexithymia. As hypothesised, the AN group were found to score significantly higher on a number of personality disturbances. However, the personality constructs in which groups significantly differed were not entirely as expected.

As predicted, the AN group displayed greater scores on obsessive compulsive, avoidant, dependent and borderline personality disorders. In addition, AN participants also scored significantly higher on schizoid, negativistic and depressive personality disturbances, which is consistent with a number of characteristics reported in individuals with AN such as reduced interest in friends and activities, and depressive symptoms [15, 23].

There were no significant correlations identified between any of the different personality dimensions and eating disorder symptomatology, or alexithymia in AN. This was not predicted, and differs to a number of studies which have suggested a relationship between personality constructs and eating disorder symptoms in AN (see [10] for a review). However, unlike the current study, other research has tended to focus on personality traits such as perfectionism and obsessionality rather than disturbances in defined personality dimensions. A number of significant relationships were, however, observed for negative mood states. Positive correlations in the AN group were found between both schizoid and avoidant personality dimensions and depression, and between borderline personality disorder scores and stress. These findings suggest that a number of personality disturbances in AN may not be related to disorder-specific symptoms, but to negative mood states / emotionality present in the disorder. One might therefore predict with the stabilisation of mood symptoms, these personality dimensions may normalise in AN.

Of particular interest is the absence of significant correlations between obsessive compulsive personality disorder and eating disorder symptomatology. AN is frequently associated with obsessive compulsive behaviours and thoughts [7, 24], and the AN group in the current study was similarly found to demonstrate increased symptoms related to obsessive compulsive personality disorder. Though these thoughts and behaviours may represent a core feature of AN, the lack of association with eating disorder symptoms suggests that these features are independent of current AN symptoms. Therefore, increased obsessive compulsive personality symptoms, and the other personality disturbances that were not influenced by negative mood state, may represent stable traits which predispose individuals to developing AN.

Although the current study has resulted in a number of important implications for our understanding of personality disorders in AN, the findings may be limited due to a number of factors. Firstly, the patients recruited for this study were recruited from a specific catchment area or were private hospital patients, thus potentially limiting the generalisability of the sample. However, AN participants were also recruited from public advertisements in an attempt to limit this potential confound. Secondly, the conservative sample size and the number of statistical comparisons undertaken have the potential to have influenced the findings. However, to accommodate this potential confound, the authors applied stringent criteria to indicate significant results, thus limiting the number of false positive errors. An additional limitation of the study is that only acutely ill AN participants were tested, thus a number of findings may reflect state rather than trait effects. As personality disturbances are often reported as a result of starvation [25], future research would benefit from also investigating long term-recovered AN patients whose body weight has restored and negative mood symptoms may have subsided, to examine the stability of personality disturbances in AN. Therefore, a larger sample size, and the inclusion of weight-restored or clinically recovered AN patients would be of benefit in future research. Furthermore, longitudinally assessing the stability of these findings across the different phases of the illness would be beneficial.

## Conclusions

Overall, the findings of the study suggest that individuals with AN exhibit a number of personality disturbances relative to healthy individuals. A number of these personality dimensions were related to negative mood state in AN, though none were related to eating disorder symptomatology or emotion processing within the self (alexithymia). Although the findings are limited by the conservative sample size, the results suggest that personality disturbances in AN may not be influenced by disorder-specific symptoms, but may be predisposing factors to developing the illness.
